# Prevalence of interstate telehealth comparing internet protocol and home address

**DOI:** 10.1093/haschl/qxaf129

**Published:** 2025-06-26

**Authors:** Kaustav P Shah, Ateev Mehrotra, Eric Bressman

**Affiliations:** Division of General Internal Medicine, University of Pennsylvania, Philadelphia, PA 19104, USA; Leonard Davis Institute of Health Economics, Philadelphia, PA 19104, USA; National Clinician Scholars Program, University of Pennsylvania, Philadelphia, PA 19104, USA; Department of Medicine, Corporal Michael J. Crescenz Medical Center, Veterans Affairs, Philadelphia, PA 19104, USA; Department of Health Services, Policy, and Practice, Brown University School of Public Health, Providence, Rhode Island 02903, USA; Division of General Internal Medicine, University of Pennsylvania, Philadelphia, PA 19104, USA; Leonard Davis Institute of Health Economics, Philadelphia, PA 19104, USA; Department of Medicine, Corporal Michael J. Crescenz Medical Center, Veterans Affairs, Philadelphia, PA 19104, USA

**Keywords:** telehealth, telemedicine, health policy, licensure, primary care, electronic health records

## Introduction

When providing telehealth, clinicians typically must be licensed in the state where the patient is located. However, early in the COVID-19 pandemic, states implemented waivers relaxing these rules.^1^ Prior estimates of the prevalence of interstate visits—∼5% of all telehealth—used patients’ home addresses in claims or electronic health records (EHRs) as a proxy for the patient's actual location.^2,3^ This approach overlooks patient travel, likely underestimating the true prevalence of interstate care.

There are ongoing policy discussions on licensure reform to support interstate telehealth. To help inform this discussion, we used geolocation data that contain patient internet protocol (IP) addresses from our academic health center's telehealth vendor to more precisely estimate interstate telehealth rates during the period where state waivers allowed for interstate care.

## Methods

We identified all adult primary care telehealth encounters (May 2020 to September 2021) in the health system's EHR and linked these to BlueJeans data, a telehealth platform that provided IP address and associated geolocation (city, state, and country) from where the patient accessed the encounter. Patient demographics and home addresses were obtained from the EHR.

A visit was considered out-of-state when the provider's practice site and the patient's location were in different states. We compared the prevalence of interstate telehealth visits using patient's IP address location vs. EHR-documented home address as markers of patient location. We compared demographic characteristics of patients with visits from multiple states and the rest of the cohort. Analyses were conducted using STATA 18. The University of Pennsylvania's Institutional Review Board determined this study exempt.

## Results

There were 94 109 patients with 150 343 primary care telehealth encounters during the study period (Table 1). Mean age was 47.8 years (SD: 17.3); 65.0% were female; and 62.2% identified as White and 23.2% as Black.

**Table 1.** Patient characteristics, stratified by whether they took visits from multiple states (or countries) during the study period.

	Total patients (*N* = 94 109)	Patients with visits from a single state (or country) (*n* = 87 870)^a^	Patients with visits from multiple states (or countries) (*n* = 6239)
Total encounters	150 343	131 018	19 325
Encounters with state discrepancy, %	12.0	7.3	44.5
Age, mean (SD), years	47.8 (17.3)	48.0 (17.4)	44.7 (16.2)
Female, *n* (%)	61 200 (65.0)	56 885 (64.6)	4315 (69.2)
Race, *n* (%)			
White	58 538 (62.2)	54 918 (62.5)	3620 (58.0)
Black	21 861 (23.2)	20 046 (22.8)	1815 (29.1)
Asian	3886 (4.1)	3711 (4.2)	175 (2.8)
Hispanic-Latino	3102 (3.3)	2872 (3.3)	230 (3.7)
Other/unknown	6722 (7.1)	6323 (7.2)	399 (6.4)
English as primary language, *n* (%)	92 807 (98.6)	86 643 (98.6)	6164 (98.8)
Number of unique states accessing encounters from			
1	87 771	—	—
2	5609	—	—
3	567	—	—
4	56	—	—
5	7	—	—

^a^Includes patients with visits from only one jurisdiction, even if another country.

The estimated prevalence of out-of-state telehealth visits for the health system was 10.1% using EHR address and 19.2% using IP address. A state discrepancy between the patient's EHR and IP address was seen in 12.0% of telehealth encounters.

Overall, 32 061 patients (34.0%) in the sample had multiple telehealth visits. Among this subgroup, 6239 (19.5%) had visits from ≥2 distinct states. Patients with telehealth visits from multiple states were younger (44.7 vs 48.0) and more often female (69.2% vs 64.7%) or Black (29.1% vs 22.8%) than single-state users.

Patients accessed encounters from every state in the United States except North Dakota and Idaho, with the highest volumes from neighboring and populous states (Figure S1). During a small number of encounters (192 or 0.12%), patients were out of the country (53 total countries).

## Discussion

In the first study to use IP addresses to estimate patients’ locations during telehealth encounters, we find nearly 1 in 5 visits were out-of-state when licensure constraints were absent. Additionally, patients rarely accessed care from outside the United States entirely. Our results indicate the true prevalence of interstate telehealth nationally is likely much higher than the 5% estimated in prior analyses^2,3^ that used home addresses.

Many patients value telehealth precisely because it enables continuity with existing providers as travel between work, school, or family may bring them to different states. Under current regulations, providers are expected to verify patients’ real-time location, although this requirement is rarely enforced. We show that relying on the patient's EHR address for verification would misidentify the patient's actual state in 12% of encounters. The high interstate telehealth rates seen here emphasize the need for physician licensure reforms to align healthcare with the realities of the digital age and modern travel.^4,5^ Proposals such as a national telehealth license or state telehealth reciprocity agreements would permit these interstate encounters and reduce barriers to care continuity without creating additional administrative burdens for providers. Licensure reform may be especially important for rural patients who often cross states for specialty care,^6^ as well as for patients who are younger, are female, or identify as Black or Hispanic, who we found had higher rates of visits from multiple states.

Limitations of this work include that the data come from early in the pandemic when travel patterns were atypical and state waivers were active; however, this allows for estimation of interstate telehealth rates without state licensure constraints; IP addresses may be masked by virtual private networks; and the system sits at a tristate border, meaning absolute out-of-state numbers may not be generalizable.

In summary, we use IP addresses to estimate high rates of interstate telehealth while licensure restrictions were waived. Policymakers should consider licensure reform as part of future telehealth policy proposals.

## Disclaimer

The views expressed are those of the authors and do not necessarily reflect the position or policy of the VA or the US government.

## Supplementary Material

qxaf129_Supplementary_Data

## Data Availability

The data underlying this article cannot be shared publicly due to the sensitivity of geolocation data.
