# Supplementary material for: Prevalence of interstate telehealth comparing internet protocol and home address
Source: Health Aff Sch. 2025 Jun 26;3(7):qxaf129. doi: 10.1093/haschl/qxaf129 (PMC12254946; doi:10.1093/haschl/qxaf129)
Supplement: qxaf129_Supplementary_Data [file qxaf129_supplementary_data.pptx]

## Slide 1
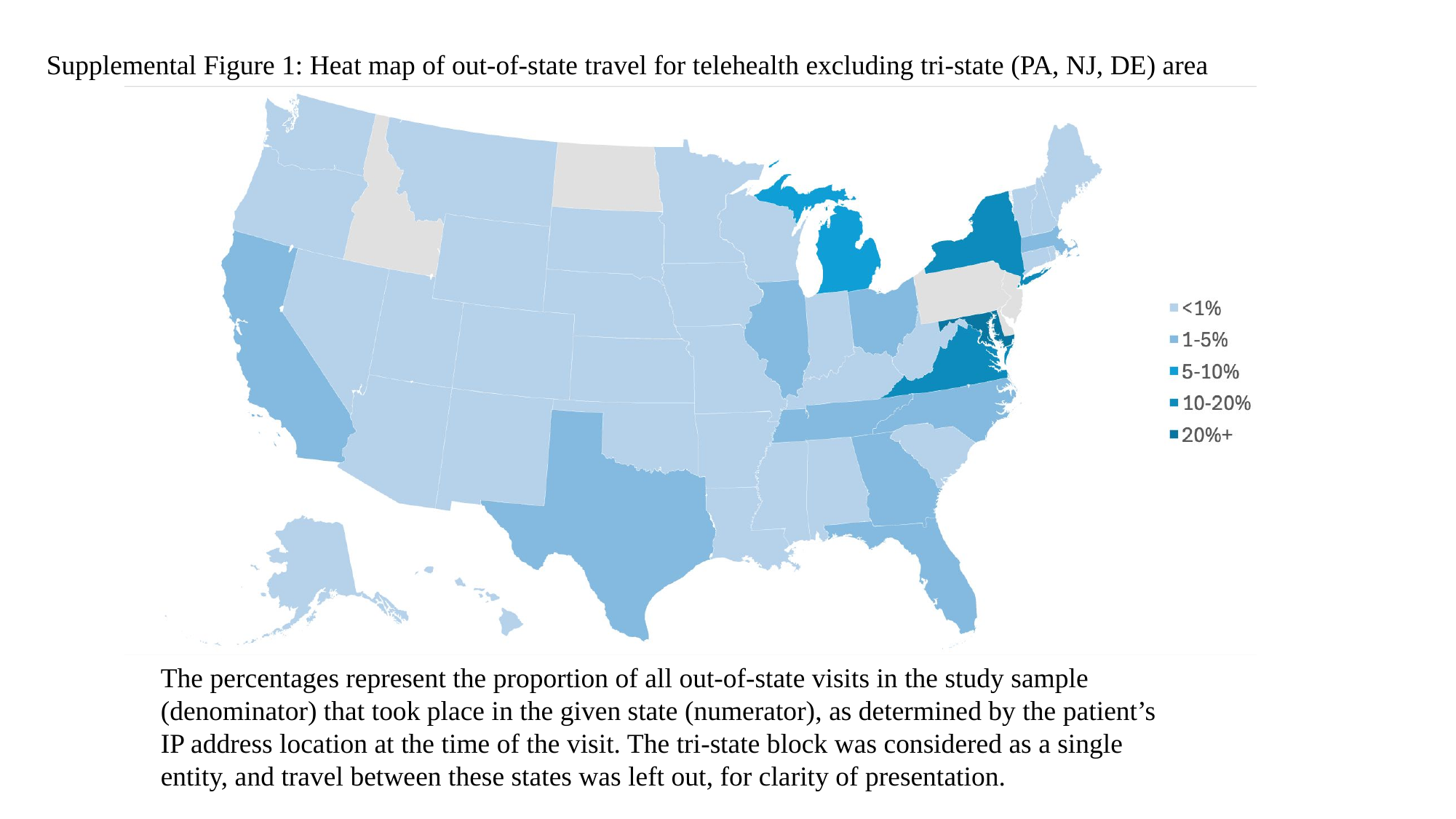

Supplemental Figure 1: Heat map of out-of-state travel for telehealth excluding tri-state (PA, NJ, DE) area
The percentages represent the proportion of all out-of-state visits in the study sample (denominator) that took place in the given state (numerator), as determined by the patient’s IP address location at the time of the visit. The tri-state block was considered as a single entity, and travel between these states was left out, for clarity of presentation.
